# Extensive Recontouring of the Femoral Head with Osteochondral Allografting: A Case Report with Histological and MicroCT Analysis

**DOI:** 10.1155/2019/6956391

**Published:** 2019-11-21

**Authors:** Amir A. Jamali, Douglas Rowland, Kristen N. Vandewalker

**Affiliations:** ^1^Joint Preservation Institute, 100 N. Wiget Lane, Suite 200, Walnut Creek, CA 94598, USA; ^2^Center for Molecular and Genomic Imaging, Animal Imaging Shared Resource, UCD Comprehensive Cancer Center, University of California, Davis, USA; ^3^Sutter General Hospital, Department of Laboratory Medicine, Sutter Medical Foundation Laboratory-Microbiology, USA

## Abstract

Morphological abnormalities such as cam deformity or growth disturbances can have a detrimental effect on the smooth function of the hip joint. This case reports an attempt to salvage the hip joint of a young patient with a posttraumatic growth disturbance of the femoral head using a fresh osteochondral allograft. This treatment has been used very rarely in the femoral head due to the presumed tenuous blood supply of the head and the perceived risk of nonunion or progressive avascular necrosis. The patient in this case had persistent pain and mechanical symptoms leading to hip replacement. A detailed analysis of the retrieved femoral head demonstrated durability and healing of the grafts based on gross inspection, histology of bone and cartilage, and microCT analysis. This case is the first report to our knowledge of a detailed histological and radiographic analysis of the fate of osteochondral allografts of the femoral head. We hope that this case provides justification for the use of osteochondral allografts of the femoral head for other indications such as femoral head fractures, avascular necrosis, and benign epiphyseal tumors of the femoral head in an effort to avoid arthroplasty in young patients. The authors have obtained the patient's informed written consent for print and electronic publication of the case report.

## 1. Case Report

**Figure 1:**
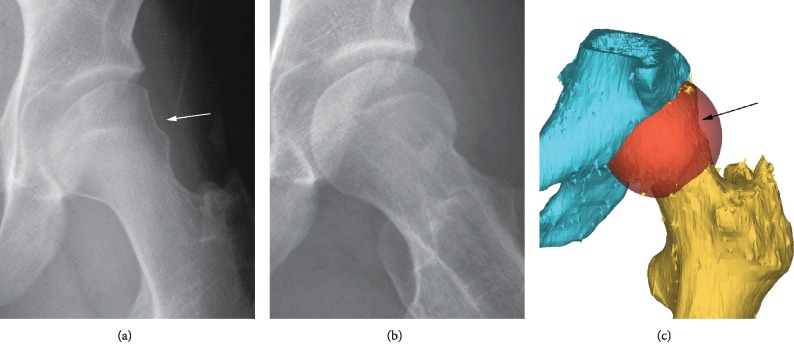
(a) Anteroposterior plain radiograph of the left hip demonstrating a femoral head deformity laterally (white arrow) with no signs of osteoarthritis. (b) Lateral plain radiograph demonstrating normal morphology of the femoral head with no signs of osteoarthritis. (c) Three-dimensional reconstruction of the pelvis (blue) and proximal femur (gold) with superimposed spherical virtual femoral head (orange) demonstrating the area of missing bone on the lateral femoral head (black arrow).

A 22-year-old male was examined for a chief complaint of left hip pain. He had sustained a left femoral shaft fracture, treated with a locked intramedullary nail placed through a trochanteric start point at the age of 13. Over the ensuing 5 years, a growth disturbance and deformity of the lateral aspect of the femoral head developed. The patient complained of pain and catching in the hip. He was treated with a diagnostic hip arthroscopy as well as limited recontouring of the head at the anterior aspect. The lateral area of head hypoplasia could not be addressed. The patient's pain continued after that procedure. A 3D CT scan was obtained, and reconstructions were generated. This demonstrated a global absence of bone on the lateral aspect of the femoral head. (Figures 1(a) and 1(b)).

Due to his age and absence of osteoarthritis, surgical hip dislocation and osteochondral allografting of the femoral head were recommended in order to address the area of lateral femoral head hypoplasia. His unexplained mechanical symptoms were thought to be a result of the morphological abnormality of lateral femoral head hypoplasia. A discussion was carried out with the patient and family that the correction of this deformity may be able to alleviate his symptoms. A three-dimensional virtual plan was created to delineate the exact area of hypoplasia in planning the allograft procedure (Figure 1(c)).

A 52 mm femoral head graft was requested based on the size of his femoral head on CT. A fresh osteochondral allograft femoral head became available from a 17-year-old male donor (Joint Restoration Foundation, Centennial, CO, USA). The graft was never frozen and was kept in tissue culture media provided by the tissue bank at 4°C during its storage and transportation. The graft procedure was scheduled exactly 7 days after the graft recovery to maximize cell viability.

## 2. Surgical Dislocation/Osteochondral Allografting Procedure

**Figure 2:**
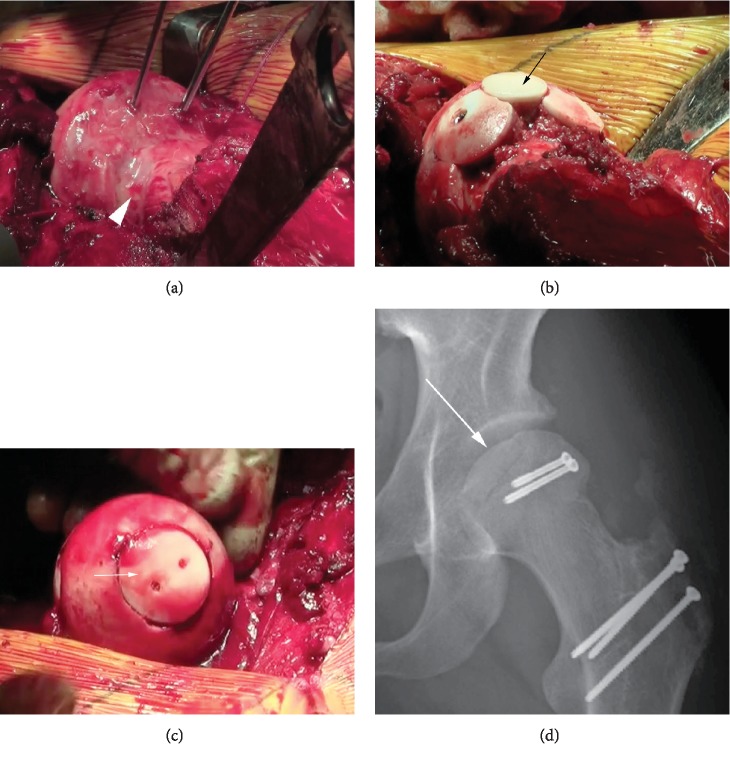
(a) Intraoperative photograph demonstrating guidepins placed in the lateral femoral head defect prior to coring for osteochondral allografting. The white arrowhead shows the anterior extent of the lateral femoral head lesion. (b) Intraoperative photograph demonstrating osteochondral allografts restoring the lateral contour of the femoral head at the side of the lateral femoral head defect. The central graft (black arrow) is in the process of being fully seated. (c) Intraoperative photograph of the femoral head apex with a 25 mm osteochondral allograft (white arrow) stabilized by two PLLA pins. (d) Immediate postoperative AP hip radiograph after femoral head osteochondral allografting through a trochanteric osteotomy with 2 metal screws holding the lateral femoral grafts in place. The apical femoral head graft is demonstrated by the white arrow.

The patient underwent surgical hip dislocation [1] with labral repair and osteochondral allografting of the femoral head. The patient was placed in the lateral decubitus position. A posterolateral approach to the hip was performed with a trochanteric flip osteotomy. The hip was exposed, opened, and dislocated according to the standard technique. A new tear was demonstrated in the previously repaired hip labrum. The labrum was repaired with three suture anchors. The femoral head still has some prominent edges anteriorly. These sharp edges were smoothened with a burr. The articular cartilage of the femoral head and acetabulum was generally healthy. The lateral synovial fold, the entry of the vascular pedicle of the medial femoral circumflex artery (MFCA), was protected. The area of lateral femoral head hypoplasia was immediately proximal to the MFCA (Figure 2(a)) with three grafts being placed proximal to the entry site of the MFCA into bone.

A large 25 mm full thickness area of cartilage loss and flattening was noted at surgery at the superior, apical aspect of the head. A total of 3 grafts were placed in the lateral femoral head and interlocked in a “snowman” configuration such that the edge of the peripheral grafts was removed to accommodate the central most graft (Figure 2(b)).

These were implanted using a widely used commercially available transplantation set (Allograft OATS, Arthrex, Naples, FL, USA). The allografts utilized in the lateral head measured 15 mm, 18 mm, and 20 mm in size. They were stablized using a total of two 3.0 mm cannulated screws (Synthes, Paoli, PA, USA). In the superior dome, a 25 mm core graft was obtained from the donor's femoral head and implanted, leading to a smooth surface of the articular cartilage (Figure 2(c)).

That graft was stabilized with two 1.3 mm polylactic acid chondral darts (PLLA Chondral Dart, Arthrex, Naples, FL, USA) (Figure 2(c)) The hip was reduced and checked with range of motion to be sure there was no catching. The capsule was repaired followed by the repair of the trochanteric osteotomy with a total of three 3.5 mm cortical screws (Synthes, Paoli, PA, USA).

## 3. Postoperative Recovery

Postoperative radiographs demonstrated restoration of a normal femoral head contour (Figure 2(d)).

The patient was kept at partial weight-bearing of 30 lbs on the affected leg for 3 months. After the procedure, he had a period of good pain relief for 6 months. However, as the patient became more active, the popping in the hip returned. Ultimately, it was felt that his mechanical symptoms were likely due to progressive femoral head or acetabular cartilage degeneration or some other source of pathology in the hip. At one year postoperatively, the patient requested treatment with total hip arthroplasty. He underwent uncemented total hip arthroplasty. At the time of arthroplasty, the femoral head was removed with the patient's consent for further analysis. The patient is now 6 years post arthroplasty and is doing well clinically with no hip pain.

## 4. Femoral Head Analysis

### 4.1. Visual Inspection

**Figure 3:**
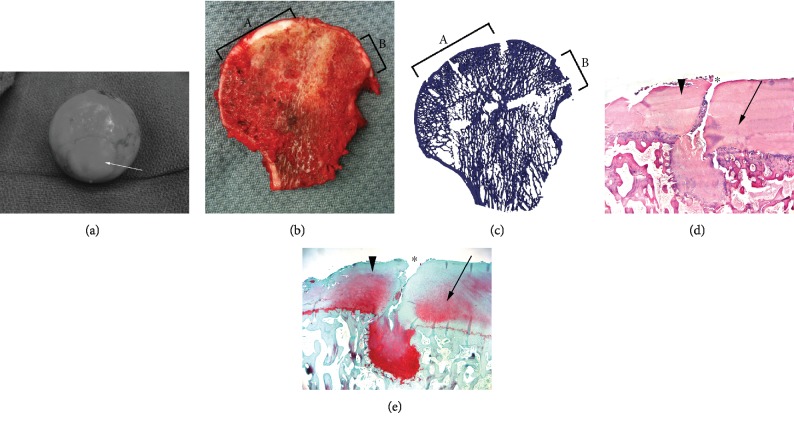
(a) Intraoperative photograph at time of total hip replacement showing full integration of the apical 25 mm allograft (white arrow) into the native femoral head. (b) Sectioned femoral head at time of surgery. The apical graft (delineated by “A”) and central lateral graft (delineated by “B”) are included in this section. (c) MicroCT section of femoral head corresponding to (b). The apical graft (delineated by “A”) and central lateral graft (delineated by “B”) are included in this image. (d) Hematoxylin and eosin section of interface showing the native femoral head (large black arrowhead), apical femoral osteochondral allograft (small black arrow), and interface fissuring seen (^∗^). (e) Safranin-O section of interface showing the native femoral head (large black arrowhead), apical femoral osteochondral allograft (small black arrow), and interface fissuring seen (^∗^). This section demonstrates presence of slightly decreased proteoglycan content in the allograft section.

On visual inspection, one could see the position of the grafts. There was no evidence of cyst formation, cartilage loss, avascular necrosis, or osteoarthritis on surface inspection (Figure 3(a)).

The retrieved femoral head was sectioned in the operating room (Figure 3(b)) to include portions of both the apical graft and the central lateral femoral head graft.

### 4.2. MicroCT

The femoral head slice was imaged at the Center for Molecular and Genomic Imaging at UC Davis with X-ray CT (Figure 3(c)).

The slice was kept in a plastic bag to retain moisture and mounted to a custom rod for imaging. X-ray tomographic images were obtained on the Centers MicroXCT-200 specimen CT scanner (Carl Zeiss X-ray microscopy). The sample was mounted on the scanners' sample stage, which has submicron level of position adjustments. Scan parameters were adjusted based on the manufacturer's recommended guidelines. The source and detector distances were set at 108 mm and 30 mm, respectively. Once the source and detector settings were established, the optimal X-ray filtration was determined by selecting among one of 12 proprietary filters; in this case, the high-energy (HE2) filter was chosen. Following this procedure, the optimal voltage and power settings were determined for optimal contrast (40 kV and 200*μ*Amp). 2600 projections over 360 degrees were obtained with 2 seconds per projection. The camera pixels were binned by 2, and the source-detector configuration resulted in a voxel size of 53 *μ*m. The tomographic image was reconstructed with a center shift (-0.1 pixels) and beam hardening parameter value of 0.1 to obtain optimized images. A smoothing filter of kernel size 0.7 was applied during reconstruction. Images were reconstructed into 16 bit values. On microCT examination of the same section shown in Figure 3(b), the bone surface was congruent with no signs of collapse at the site of the grafts or at the graft/host interface. There was no evidence of avascular necrosis.

### 4.3. Histological Analysis

Histological analysis was performed after decalcification. The tissue was then analyzed using hematoxylin and eosin (Figure 3(d)) staining and Safranin-O staining (Figure 3(e)).

There was a persistent fissure at the interface of the donor and recipient cartilage, a finding that has been shown previously in osteochondral grafts of the knee [2]. The cell viability in the grafts appeared to be normal with no signs of apoptosis or other forms of necrosis. The cartilage of the grafts had a normal columnar architecture. The cartilage surface was intact on both the grafts and the surrounding host bone indicating no sign of osteoarthritis or avascular necrosis.

## 5. Discussion

The case presented here illustrates the challenges in dealing with morphological abnormalities of the femoral head. In this case, the patient had a presumed growth disturbance of the femoral head leading to a misshapen femoral head. The objective of this report is to demonstrate based on histology and advanced imaging that osteochondral allografting is an appropriate and advantageous treatment in cases of structural abnormalities of the femoral head from trauma, avascular necrosis, or osteoarthritis.

The femoral head has been viewed by some as a “no man's land” for cartilage treatment. This has largely been a result of the lack of access to the femoral head due to its tenuous blood supply [3, 4]. This concern has largely been addressed through the work of Ganz and colleagues in developing the surgical hip dislocation procedure with careful preservation of the vascular supply to the femoral head [1]. The main remaining challenge is to determine whether osteochondral allografting is a viable solution in the treatment of the femoral head for articular cartilage lesions and osteochondral defects caused by trauma or avascular necrosis. Most experts seeking to avoid total hip replacement for their patients with femoral head avascular necrosis have utilized a number of approaches including allograft and autograft fibular struts, progenitor cell transplants, and trap-door procedures [5–10]. There have been scattered case reports of osteochondral allografts for femoral head lesions. Since avascular necrosis has been a concern, limiting the use of osteochondral grafts in the femoral head, we sought to prove that one could achieve excellent joint surface restoration using fresh osteochondral allograft transplantation in this anatomical area with full healing of the interface between the osteochondral allografts and the native femoral head.

In this case, we were able to perform osteochondral allografting of a substantial portion of the femoral head using the surgical dislocation approach. The gross appearance, microCT, and histological evidence suggest that the procedure achieved its goals of restoring the femoral head to a normal structure without complications such as graft failure, collapse, necrosis, or other issues. In spite of the technical success, the patient continued to complain of pain and popping in the hip. This ultimately led to the need for total hip replacement.

A number of case reports and other publications have detailed the treatment of femoral head cartilage lesions and fractures with osteochondral allograft transplants. These procedures have been performed for conditions such as hip dislocation [11], tumors [12, 13], osteochondritis dissecans (OCD) [14], and avascular necrosis [14–16]. Myers reported on the first series of osteochondral allografts of the femoral head, 25 hips with a diagnosis of avascular necrosis (24 hips) or femoral head fracture (1 hip) [15]. Of the hips with steroid-dependent AVN (10 hips), 50% failed while among the cases of posttraumatic, alcohol-related, and idiopathic AVN (14 hips), only 21% failed. Kosashvili et al. reported on 8 hips treated with fresh osteochondral allografting of the femoral head between 2008 and 2010, 4 with OCD, 3 with avascular necrosis, and one with a femoral head fracture [17]. Five had a good to excellent result, one was converted to total hip replacement, one underwent revision allografting, and one required removal of hardware. In this series, the worst results were in steroid-induced avascular necrosis. The authors performed a follow-up report encompassing 17 patients (including the 8 from the first series) and showed that 13 had fair to good outcomes [14]. In this group, there were a total of 3 failures requiring arthroplasty and one requiring revision allografting. The authors did point out that in the more up to date series, two patients with steroid-induced AVN were both doing clinically well at latest follow-up (one at 24 months and one at 3 months).

Our analysis revealed that much like osteochondral allografts of the knee and ankle, femoral head grafts demonstrated consistent healing across the host-graft interface based on visual inspection, histological analysis, and microCT analysis. We further demonstrated maintenance of cartilage histology based on Safranin-O analysis.

We hope that this work and the work of other groups that have explored osteochondral allografts will continue to push for this treatment as a potential treatment option in cases of isolated femoral head collapse or extensive articular cartilage injury of the femoral head in an otherwise healthy hip joint, particularly in the young patient.
